# Round the Hospitals

**Published:** 1917-03-24

**Authors:** 


### ROUND THE HOSPITALS.

A claim has been advanced to every Member of Parliament that the Society for the State Registration of Trained Nurses is composed of 4,000 certificated nurses, with the request that each member will use his influence to secure that, before any Bill for the State Registration of Trained Nurses is promoted by the Government, what are described as the organised societies of nurses in England, Scotland, and Ireland—*i.e.* organised by the Society in question—may be permitted to express "their opinion concerning the organisation of their profession." Members of Parliament are entitled to the information that as a matter of precaution it may be well that every statesman amongst them should request to have the names and addresses of the 4,000 certificated nurses in question supplied to him. He will then be in a position, as a public man, before taking any step in compliance with the request, to ascertain the actual facts. This action seems desirable from the circumstance that within our knowledge some of the most active supporters of the Royal College of Nursing are members of the Society for the State Registration of Trained Nurses, and recently other of the Society's members have applied for admission to the Register of the College. The recruiting for membership of the Society for the State Registration of Trained Nurses has been carried on for a number of years, long before the Royal College of Nursing came into existence or Mr. Stanley's proposals were formulated. It is fair to conclude that, if 4,000 is the total number of members on the books of the Society to-day, not a few of them may have changed their minds or joined the College, or ceased to have sympathy with the prayer of the letter which has been addressed by the President to Members of Parliament. It is creditable to the President that she should use the phrase "4,000 certificated nurses of which this Society is composed." Such language may satisfy each Member of Parliament that caution is called for, and that evidence should be sought of the exact numbers behind the President to-day, that members of the House of Commons may know what proportion the present members of the Society for the State Registration bear to the total number of British nurses, who probably amount in the whole to at least 60,000 fully trained and certificated nurses.

---

Members of Parliament are entitled to learn from the President, too, how many members of her Society hold that each of these gentlemen who were educated at Eton, Harrow, Winchester, Rugby, or some other great public school, or is a member of one of our great universities, has received his education *in forma pauperis*, seeing that the President claims that to raise an endowment fund for the Royal College of British Nursing has aroused the self-respecting element in the nursing profession—which appears to be confined to members of the Society for the State Registration of Trained Nurses—each of whom "naturally bitterly resents being thus utilised *in forma pauperis* in support of the College scheme." Truly, there are few things reason can discover with so much certainty and ease as its own insufficiency.

---

We are glad to find that the attention we are giving to nursing problems in Ireland is becoming more appreciated each week. We have been thanked for the article on "Registration: its Limitations and Plan of Campaign," which appeared on page 483 of last week's The Hospital. That article has received especial welcome and has, already, we are assured, done something to promote united action and pave the way to a solution of the difficulties at present dividing Irish opinion in regard to the Royal College of British Nursing. There is a strong desire on the part of independent Irish nurses

**ROUND THE HOSPITALS—(*continued*).**

who are qualified to register with the College that care shall be taken that no dallying, like that which shut the Irish nurses out of the advantages enjoyed by English nurses through the Midwives Act, must be allowed to exclude them from the College, its Register, Educational Advantages and Prestige. Important developments may be looked for in Ireland shortly. We hope that the solution of Ireland's difficulties in regard to the Royal College of British Nursing and to those which delay a satisfactory settlement by agreement, may be arrived at, simultaneously with the solving of Ireland's political perplexities.

---

As we expected, the Manchester Branch of the National Union of Trained Nurses has taken further action, and is seeking the co-operation of the members of all other branches of the Union in support of its protest against the policy of the Executive as summarised by the honorary secretary, who is not a fully trained nurse, "to fight the College to the death"—that is, to employ the Union organisation to promote disunion amongst members of the nursing profession. The Manchester Branch has sent out to 355 members the following resolution: "That the Manchester Branch very much regrets the action of the Council in opposing the College of Nursing in its endeavours to obtain union, and begs the Council of the National Union of Trained Nurses to change its policy, and, if possible, to co-operate with the College." Two hundred and ten members voted, of whom 202 were in favour of and only five against the resolution. A copy of the resolution and the result of the voting have been sent to the Executive Committee of the Union and also to the Branch Secretaries, asking them to lay the matter before their committees and members. We hope this action may be the means of moving the independent members of the Union to combine with the Manchester Branch in united action to re-establish and enforce the original policy with which the Union started—namely, to uphold and maintain the highest ideals and truest objects for the welfare of trained nurses.

---

IT was evident that the whole of the vast audience at the Women's National Service meeting at the Albert Hall on Saturday, March 17, were filled with loyal pride and deep reverence for the Queen because of Her Majesty's truly patriotic action in putting aside the indulgence of private grief in order to be present at the great national gathering. The Queen and almost all the other ladies present were, of course, habited in black, but a welcome note of colour was supplied by what might be called the supporters of the Royal box. On one side were a number of military nurses in their grey uniform and red facings, while the corresponding box on the other side was occupied by naval nurses from Chatham, in their blue gowns and red facings, while their head-coverings were dainty snow-white coifs, with a Royal crown embroidered in blue. Nursing received its full meed of justice from the speakers. Lord Derby spoke of the priceless work done not only by the regular nurses but by those of the V.A.D.s; and Miss Violet Markham dwelt on the need for district nurses. Mrs. Tennant showed the other side of the present situation when she mentioned that the Care Committees of the London County Council had lost nearly half of their 9,000 workers—lured away, perhaps, by the glamour of what appeared to be more definitely war work. Both ladies spoke strongly in favour of the work of child welfare and the needs of mothers, and gently deprecated the desertion of such obviously womanly tasks in favour of other occupations. Mrs. Tennant made a suggestion that will be welcome to many women who are conscious that they can do but little to help in the winning of the war, when she recommended the saving and collecting of every scrap of wool and cotton which can be re-manufactured for use. Mr. Prothero, in inviting women to come into agriculture, warned them that their lot would be no easy one, but like the rest of the speakers he realised that the idea of sacrifice was of itself an attraction to women—an idea to which Mr. Hodge gave breezy expression when he said that at Mr. Prothero's announcement he expected to hear the women present burst into some such song as "Whistle and I'll come to you, my lad." From the attention which was accorded to each speech it was evident that every woman present—not only those in the audience but the khaki-clad members of the Women's Volunteer Reserve who acted as stewards, and the white-robed members of the choir who beguiled the long waiting before the meeting with songs, were keen to do all they could to help their country in its great days of trial and to bring the war to a triumphant conclusion.

---

THE *British Journal of Nursing*, which insistently proclaims itself "the only professional nursing journal," lays it down that "you cannot teach what you do not know, and the laity have no more right to teach the science of nursing or to voice the opinions of our profession than they have to assume the same responsibility towards the medical profession. Under lay control commercial influence is paramount. We may leave it at that." It follows that we may take it up at that, and what do we find? That the *British Journal of Nursing* is, like its contemporaries, a lay and commercial journal, using the words "lay" and "commercial" in the same sense as they are applied in its columns to its contemporaries in the nursing press. It is owned by a company, The Nursing Press Limited, with a nominal capital of £10,000, divided into 1,500 ordinary shares and 500 preference shares of £5 each. It was sold to The Nursing Press Limited by Mrs. E. G. Fenwick, under an agreement dated May 30, 1906, for £7,500 nominal in 1,500 ordinary shares of £5 each, not for cash, but as fully paid shares. In order to constitute it a limited company the Memorandum and Articles of Association were signed by seven laymen, that is by two chartered and one incorporated accountant, a gentleman, an articled clerk, a secretary to a public company, and a clerk. Thus, the first shareholders in the journal which claims to be "the only professional nursing journal" were laymen, that is to say, the steps taken in forming The Nursing Press Limited were identical with those taken by the Hon. Arthur Stanley at the formation of the College of Nursing Limited, with this important difference, that the signatories to the College Memorandum and Articles of Association included Mr. Arthur Stanley as the responsible organiser and the person to whom the Nursing Profession, Parliament, and the Public could look as a guarantee for its disinterested, impartial, and statesmanlike constitution. All the ink which the Editor of the *British Journal of Nursing* has expended in abusing the College because of the seven laymen who signed its Articles of Association, the nursing profession, the press, members of Parliament and all impartial people will perceive might with equal justice have been used in the abuse of the Company which owns the *British Journal of Nursing*.

The list of shareholders shows that the shares taken by the seven laymen have been transferred to "A. Bedford Fenwick." The nominal capital of The Nursing Press Limited is £10,000 in 1,500 ordinary and 500 preference shares of £5 each. The 1,500 ordinary shares were allotted as fully paid up, of which 1,200 were held in July, 1906, by "Bedford Fenwick, consulting physician"; 280 by "E. G. Fenwick, a married woman"; and 20 by "Margaret Breay, a trained nurse." One hundred and seventy of the 500 preference shares of £5 each were allotted for cash, of which 163 were taken up by fifty-eight trained nurses and seven by "Bedford Fenwick, "consulting physician." On July 11, 1916, 176 preference shares had been allotted, and £5 per share had been paid upon each of them in cash. These 176 shares were held on the date in question by fifty trained nurses, one spinster, and "Bedford Fenwick, consulting physician," whose holding of preference shares had increased to twenty-six.

Each member is entitled to one vote for every share. The preference shares are entitled to 5 per cent., and then the ordinary shares take 8 per cent. of the profits. The control of the company is in the hands of Dr. and Mrs. Bedford Fenwick, who at the date of the return of 1916 held together 1,480 ordinary shares and twenty-six preference shares. The whole of the cash payments appear to have been made in respect of 176 preference shares which are held by shareholders, each of whom is described, with only two exceptions, as "trained nurse." These facts demonstrate the substantial humbug of the *British Journal of Nursing's* contention that it is "the only professional nursing journal" in the sense in which it uses the words. May we hope that the policy of make-believe pursued for so many years by this journal, and its misuse of the terms "laymen," "pseudo-nursing," and "commercial press," whatever may be the precise meaning these terms possess in the minds of the Editor and proprietors of the *British Journal of Nursing*, will in future be applied by both to that paper and to its nursing contemporaries, impartially, seeing that all of them appear to rest upon business foundations which are mainly identical?

